# Healthy aging index and its link with relative education between individual and neighborhood: a population-based, cohort study

**DOI:** 10.1186/s12877-022-03469-7

**Published:** 2022-10-03

**Authors:** Chunyu Lu, Jingru Zong, Lingli Wang, Yajie Du, Qing Wang

**Affiliations:** 1 Department of Biostatistics, School of Public Health, Cheeloo College of Medicine, Shandong University, 44 Wenhua West Road, Jinan, 250012 Shandong China; 2 National Institute of Health Data Science of China, Shandong University, Jinan, 250012 Shandong China

**Keywords:** China, Healthy aging index, Neighborhood, Education, Person–neighborhood fit

## Abstract

**Objectives:**

There is increasing recognition of the importance of neighborhood socioeconomic status (SES) for establishing an age-friendly society. Despite the benefits of improved neighborhood SES, little is known about the link of relative education between individuals and neighborhoods with healthy aging. This study aims to construct a healthy aging index (HAI) accounting for indicators’ interlinkages and to test the association of the HAI with relative education between neighborhoods and individuals.

**Methods:**

The study used data from the China Health and Retirement Longitudinal Study from 2011 to 2018, including middle-aged and older adults (≥ 45 years). The final sample comprised 11633 participants residing in 443 neighborhoods with 34123 observations. Based on 13 health indicators, a hybrid method integrating network analysis with TOPSIS was applied to construct a HAI accounting for health interlinkages. Weighted multilevel linear and ordered logistic models were used to estimate the effects of neighborhood education.

**Results:**

Among the 11633 participants (mean [SD] age, 58.20 [8.91] years; 6415 women [52.82%]), the mean (SD) HAI was 48.94 (7.55) at baseline, showing a downward trend with age. Approximately 10% of participants had a HAI trajectory characterized by a low starting point and fast decline. A one-year increase in neighborhood education was independently associated with a 0.37-point increase (95% CI, 0.23–0.52) in HAI. Regardless of individual education, each participant tended to gain benefits from a neighborhood with higher education. However, the effects of increased neighborhood education were weaker for individuals whose education was lower than the neighborhood average.

**Conclusions:**

The HAI is an interaction system. Improving neighborhood education was beneficial to healthy aging, but individuals with lower education relative to the neighborhood average may experience poor person–environment fit and obtain fewer benefits from improved neighborhood education. Thus, in the process of improving neighborhood SES, individual-based interventions should be conducted for individuals whose education level is lower than the neighborhood average to achieve person–environment fit.

**Supplementary Information:**

The online version contains supplementary material available at 10.1186/s12877-022-03469-7.

## Introduction

For the first time in history, most people can expect to live into their 60s and beyond [1–3]. However, increased longevity does not equate to aging in good health [4]. In response, the World Health Organization (WHO) has set the goal of healthy aging and requires a community-wide effort to create more age-friendly environments based on the person–environment fit theory [5–7].

The person–environment fit theory highlights the degree to which older people’s capacities match the characteristics of their environments. This fit can increase their confidence in managing challenges within their environments and reduce their living stress, which, in turn, improves their overall well-being [8]. The concept of healthy aging and the age-friendly environments framework have been proposed as a guide to address aging issues, but the qualitative discussion still requires quantitative evidence to bridge practice and aging issues.

A universal set of interconnected health indicators has been pooled to quantify the extent of healthy aging, but previous indicators failed to capture the multidimensional conceptualization of health and the interlinkages between these indicators [4, 9, 10]. Healthy aging assessment starts with disease-based conceptualizations [11–13], which have been criticized for their narrow biomedical focus. Following the definition of healthy aging proposed by the WHO, function-based approaches have been developed, and the indicators of healthy aging are extended to include freedom from disability, high physical functioning, sound mental health conditions, freedom from sensory limitations, subjectively rated health, and sound social health [3, 5, 14–16]. These approaches tend to conceptualize healthy aging as a cumulative score based on the total number of deficits present in a person, leaving open the question of measuring health interlinkages [11–15].

There is increasing recognition of the importance of neighborhood socioeconomic status (SES) for the establishment of an age-friendly society [17] with the vast majority of older adults residing in community settings and increasing intention to remain in their homes and communities as they age [17]. Some empirical studies have examined the person–environment fit by testing the joint association of individual and neighborhood SES with health [18, 19]. Despite the benefits of improved neighborhood SES, previous literature found that the effects differed by individual education, and the results were mixed. For example, Boylan and Robert [20] documented that higher neighborhood SES was associated with better cardiovascular health for those of lower, not higher, individual SES in United States while Guo et al. [21] reported that older people with lower SES living in higher-SES neighborhoods had worse health outcomes except for cancer in Hong Kong. This divergence may be because previous empirical studies failed to consider health disparities resulting from relative SES between individuals and neighborhoods. For example, as a neighborhood SES improves over time, residents may find themselves lagging behind the neighborhood average, which, in turn, could negatively affect residents’ health. Previous literature simplified the complexity of how neighborhood SES and individual SES interact and, thus, failed to capture some characteristics of the person–environment fit in healthy aging.

To fill these gaps, this study collected information on 16 deficits from middle-aged and older Chinese adults and applied a hybrid method to construct a healthy aging index (HAI) accounting for interlinkages between these health indicators. Subsequently, the separate and joint association of individual and neighborhood education with individual-level HAI at one time and the trajectory of healthy aging were estimated to reveal the person–environment fit in age-friendly environments. Health differences in relative education between individual and neighborhood were assessed as a focus, helping identify those more likely to benefit from improved neighborhood SES. Education was used to measure SES since education is not affected by health in older age and its association with HAI is less likely to be biased by reverse causality [22, 23]. Based on our results, the study may have implications for proposing environmental interventions in healthy aging throughout the aging process.

## Methods

### Study population

Data were collected from the China Health and Retirement Longitudinal Study (CHARLS), a nationally representative longitudinal survey of community-living adults aged 45 and over in China [11]. The CHARLS sample was obtained via multistage stratified probability proportional to size sampling design. A more detailed description of the study design and sampling procedure can be found in the cohort profile of CHARLS [24]. The CHARLS program received ethical approval from the Peking University Institutional Review Board (IRB00001052-11015). All participants in the CHARLS provided written informed consent. We confirmed that all methods in our study were carried out in accordance with relevant guidelines and regulations.

**Fig. 1:**
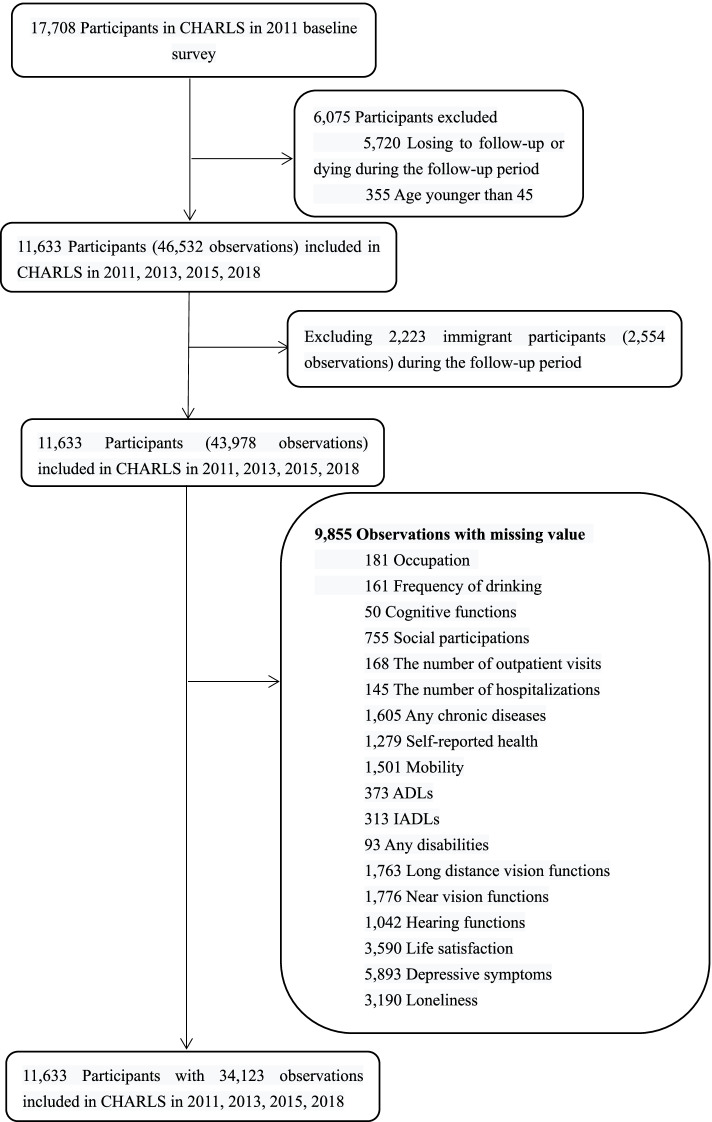
Flowchart of participant selection

In the national baseline survey in 2011, 17708 participants were interviewed. Three follow-up interviews were conducted in 2013, 2015, and 2018 with corresponding panel response rates of 88.3%, 87.15%, and 86.46%, respectively. A total of 11633 participants were interviewed in the four waves. After excluding 9855 observations with missing values and 2554 lack of neighborhood education for immigrant participants, the final sample was 11633 participants residing in 443 neighborhoods with 34123 observations. For details, see the flowchart in Fig. 1. Multiple imputation (MI) with chained equations was used to impute any missing data by creating 10 imputed datasets [25, 26] (see Supplementary Text 1).

### Individual HAI

Following the definition of healthy aging [6], 16 health measurements were included as the indicator pool for the HAI. After factor analysis and hierarchical cluster analyses, the study defined individual HAI using 13 health indicators divided into four dimensions: wellbeing (e.g., cognitive functions, social participations), physical and functional health (e.g., number of chronic diseases, activities of daily living [ADLs], instrumental ADLs [IADLs], number of disabilities, mobility), sensory health (e.g., long distance and near vision functions, hearing functions), and mental health (e.g., depressive symptoms, life satisfaction, loneliness). Interlinkages between health indicators were evaluated to represent the relative importance in individual HAI through the eigenvector centrality of each health indicator using network analysis. Using the relative importance in the HAI as weights, technique for order preference by similarity to an ideal solution (TOPSIS) was used to calculate individual HAI [27, 28]. The HAI ranged from 0 to 100 with a higher score representing greater healthy aging. Details of the construction and result of the HAI are provided in the Supplementary Texts 2 and 3.

### Individual education and neighborhood average education

Individual education was measured by a question referencing the highest level of education completed (not including adult education). It was recorded as the years taken to complete the education degree and used as a continuous variable in regression analyses. Neighborhood average education was measured by the average years of schooling for participants living in the neighborhood. The present study not only used administrative boundaries to characterize neighborhoods, like most previous studies, but also considered the social dimension of the neighborhoods (e.g., the nature of the interactions that transpire within their confines) [18, 29, 30]. A neighborhood was defined as an administrative village in a rural area and as a community in an urban area with one resident committee, where neighbors had similar access to public resources [29]. Moreover, in Chinese neighborhoods, especially in rural areas, neighbors are often relatives or old friends, frequently interacting with and helping each other [15].

Because basic education system is widely used to measure education development, we defined relative education between individual and neighbor based on China’s basic education system. Basic education consists of pre-school education, primary (6 years) and junior (9 years) and senior (12 years) middle school. As People's Republic of China was founded in 1949, the illiteracy rate is as high as 80%. In 1950, the Chinese government promoted literacy education nationwide to gradually reduce illiteracy [14]. The standard of literacy education is to have preliminary reading, writing, and arithmetic skills. Thus, we also included other two levels as no formal education, and no formal education but can read and write. In sum, according to China’s basic education system, neighborhood and individual education were attributed at five levels: no formal education, no formal education but can read and write, primary school, junior middle school, and high middle school/vocational school or above. The samples were then divided into three groups according to individual and neighborhood differences in education: individual education equal to, lower, or higher than the neighborhood’s.

### Statistical analysis

To estimate the association between neighborhood education and individual HAI, a multi-level linear model was applied. Of primary interest were the different combinations of neighborhood and individual education (e.g., individual education + neighborhood education; individual education + individual and neighborhood differences in education; neighborhood education + individual and neighborhood differences in education). The majority indicators in the final sample is comparable to the full sample of CHARLS according to their descriptive statistics. However, older people are more likely to drop out. Thus, longitudinal weights were applied in the regression analysis to correct for attrition bias. Because over 2000 observed individuals migrated from their original address to other neighborhoods during the survey period, the final weights further accounted for the migrant probability. The inverse probability weight factor was calculated by the inverse predicted probability of migration. Covariates included age, gender, marital status, area of residence of the participant (urban vs. rural), occupation, and health behavior (frequency of drinking). Occupation categories were coded from self-reported job descriptions. According to Erikson and Goldthorpe and Portocarero Class Categories, occupation was compressed to the following five categories for this study: managers and professionals, self-employed, agricultural workers, manual workers, and unemployed, with unemployed as the reference group [31]. Those who had drunk in the past 12 months were identified as “current drinker” and asked further questions about: frequency of drinking. Frequency of drinking were classified as drink more than once a month, drink but less than once a month, and none. Coefficients (Coef.) with 95% confidence intervals (CIs) were reported.

Subsequently, group-based trajectory modeling (GBTM) was applied to identify the development of the HAI across the whole life process at the individual level [32]. Using GBTM, the HAI trajectories for all respondents were classified into four groups: low starting point and fast decline, median starting point and slow decline, high starting point and slow decline, and high starting point with increase followed by decline. These reflect the temporal variation in the HAI from 2011 to 2018 (Supplementary Text 4). Then, a weighted multi-level ordered logistic model was used to investigate the association, adjusting for similar covariates. Odds ratios (ORs) with 95% CIs were reported. As robustness checks, we selected a subsample of residents who had lived in the same neighborhood for more than 15 years and then reran our regression to correct the selection bias that respondents may choose habitations due to health preference [18, 25]. Additionally, age-stratified analysis was conducted. Statistical analyses were performed using STATA 16.0.

## Results

### Descriptive characteristics

**Fig. 2:**
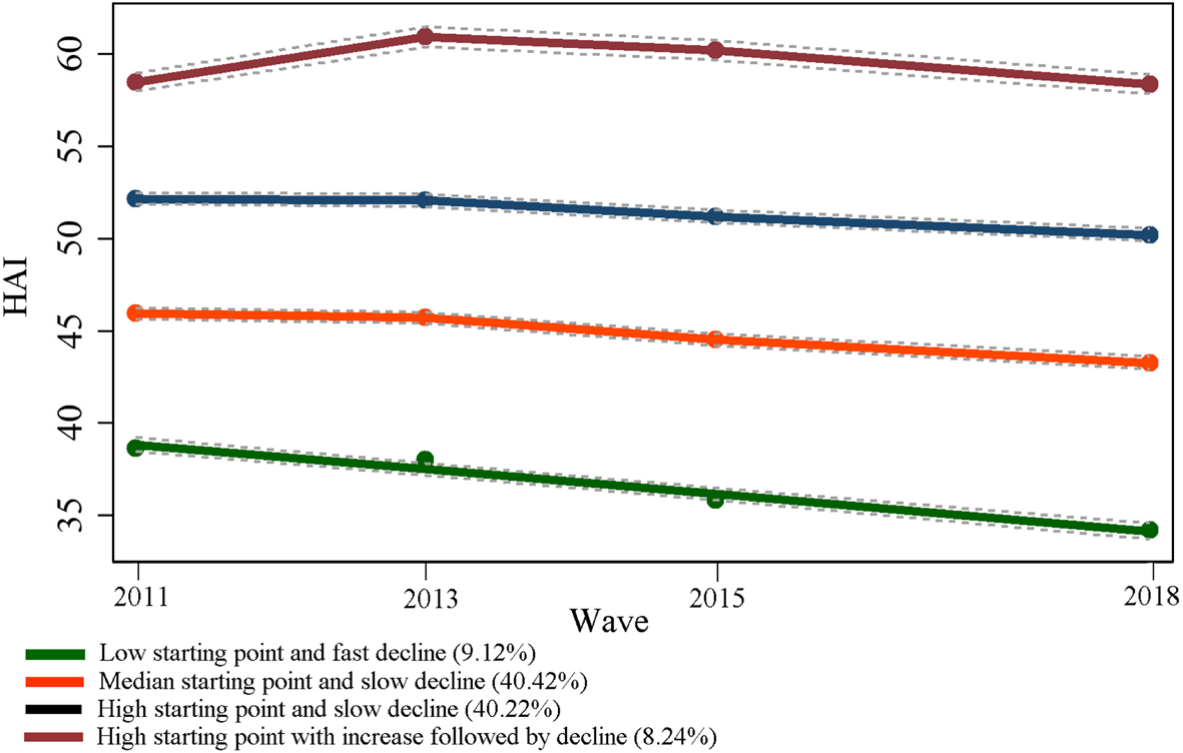
HAI trajectories. *Notes:* HAI, healthy aging index

Table 1 shows the descriptive statistics. The mean (SD) age of the 11633 participants was 58.20 (8.91) years at baseline with 6145 women (52.82%). Process results to construct the HAI are presented in Supplemental Text 3. The mean (SD) HAI was 49.26 (7.51) at baseline. The changes in the HAI were attributed primarily to mobility disabilities, which had the greatest contribution to individual HAI. Forty-two percent of participants experienced a high starting point and slow decline (Fig. 2).

**Table 1:** Baseline descriptive statistics of variables

	No. (%)				
**Characteristics**	Baseline (*n* = 11633)	Difference between individual and neighborhood education			*P* value
		Ind. Edu. = Neighbor. Edu. (*n* = 3222)	Ind. Edu. < Neighbor. Edu. (*n* = 2952)	Ind. Edu. > Neighbor. Edu. (*n* = 5459)	
**Women**	6145 (52.82)	1893 (58.75)	2277 (77.13)	1975 (36.18)	< 0.001^a^
**Age, mean (SD)**	58.20 (8.91)	59.38 (8.59)	61.61 (9.23)	55.65 (8.12)	< 0.001^b^
**Urban area of residence**	4044 (34.76)	1027 (31.87)	1014 (34.35)	2003 (36.69)	< 0.001^a^
**Individual education: years of schooling, mean (SD)**	4.89 (4.15)	3.01 (2.33)	0.69 (1.33)	8.28 (3.06)	< 0.001^b^
**Individual education**					< 0.001^a^
No formal education	3240 (27.85)	935 (29.02)	2305 (78.08)	0	
No formal education but can read and write	2157 (18.54)	1155 (35.85)	599 (20.29)	403 (7.38)	
Primary school	2507 (21.55)	989 (30.70)	48 (1.63)	1470 (26.93)	
Junior middle school	2421 (20.81)	143 (4.44)	0	2278 (41.73)	
High middle school/vocational school or above	1308 (11.24)	0	0	1308 (23.96)	
**Neighborhood education: years of schooling, mean (SD)**	4.81(1.88)	4.47 (2.00)	4.91 (1.49)	4.97 (1.96)	< 0.001^b^
**Neighborhood education**					< 0.001^a^
No formal education	1801 (15.48)	935 (29.02)	0	866 (15.86)	
No formal education but can read and write	5355 (46.03)	1155 (35.85)	1720 (58.27)	2480 (45.43)	
Primary school	4033 (34.67)	989 (30.70)	1147 (38.86)	1897 (34.75)	
Junior middle school	444 (3.82)	143 (4.44)	85 (2.88)	216 (3.96)	
High middle school/vocational school or above	0	0	0	0	
**Occupation**					< 0.001^a^
Unemployed	1985 (17.06)	599 (18.59)	570 (19.31)	816 (14.95)	
Agricultural workers	7137 (61.35)	2004 (62.20)	1796 (60.84)	3337 (61.13)	
Self-employed	905 (7.78)	218 (6.77)	225 (7.62)	462 (8.46)	
Managers and professionals	1606 (13.81)	401 (12.45)	361 (12.23)	844 (15.46)	
**Unmarried**	2302 (19.79)	643 (19.96)	578 (19.58)	1081 (19.80)	0.933^a^
**Frequency of drinking**					< 0.001^a^
Drink more than once a month	2975 (25.57)	738 (22.91)	408 (13.82)	1829 (33.50)	
Drink but less than once a month	913 (7.85)	245 (7.60)	174 (5.89)	494 (9.05)	
None	7745 (66.58)	2239 (69.49)	2370 (80.28)	3136 (57.45)	
**HAI, mean (SD)**	48.94 (7.55)	47.99 (7.19)	46.49 (7.40)	50.83 (7.34)	< 0.001^b^
**HAI trajectory**					< 0.001^a^
Low starting point and fast decline	1061 (9.12)	350 (10.86)	489 (16.57)	222 (4.07)	
Median starting point and slow decline	4720 (40.42)	1497 (46.46)	1456 (49.32)	1749 (32.04)	
High starting point and slow decline	4912 (42.22)	1204 (37.37)	917 (31.06)	2791 (51.13)	
High starting point with increase followed by decline	958 (8.24)	171 (5.31)	90 (3.05)	697 (12.77)	
**Healthy indicators**					
Cognitive functions, mean (SD)	12.04 (6.31)	11.22 (6.04)	9.30 (5.55)	14.01 (6.19)	< 0.001^b^
Social participations, mean (SD)	0.71 (0.90)	0.64 (0.81)	0.57 (0.72)	0.83 (1.01)	< 0.001^b^
Number of outpatient visits, mean (SD)	0.41 (1.41)	0.40 (1.26)	0.38 (1.34)	0.42 (1.52)	0.417^b^
Number of hospitalizations, mean (SD)	0.14 (0.59)	0.14 (0.55)	0.15 (0.72)	0.13 (0.54)	0.348^b^
Number of chronic diseases, mean (SD)	1.36 (1.39)	1.40 (1.40)	1.48 (1.43)	1.26 (1.33)	< 0.001^b^
Self-reported health, mean (SD)	3.01 (0.92)	3.00 (0.90)	3.03 (0.92)	3.01 (0.93)	0.224^b^
Mobility, mean (SD)	12.36 (4.16)	12.79 (4.30)	13.60 (4.67)	11.44 (3.51)	< 0.001^b^
ADLs, mean (SD)	6.41 (1.39)	6.50 (1.62)	6.55 (1.53)	6.28 (1.12)	< 0.001^b^
IADLs, mean (SD)	5.73 (2.01)	5.84 (2.14)	6.18 (2.52)	5.42 (1.52)	< 0.001^b^
Number of disabilities, mean (SD)	0.21 (0.52)	0.23 (0.55)	0.27 (0.57)	0.17 (0.47)	< 0.001^b^
Long distance vision functions, mean (SD)	3.69 (0.99)	3.77 (0.97)	3.87 (0.98)	3.55 (0.99)	< 0.001^b^
Near vision functions, mean (SD)	3.80 (0.93)	3.86 (0.90)	3.91 (0.92)	3.71 (0.94)	< 0.001^b^
Hearing functions, mean (SD)	3.53 (0.94)	3.57 (0.93)	3.66 (0.93)	3.43 (0.93)	< 0.001^b^
Life satisfaction, mean (SD)	2.95 (0.71)	2.96 (0.72)	2.92 (0.77)	2.95 (0.68)	0.012^b^
Depressive symptoms, mean (SD)	8.48 (6.26)	8.95 (6.34)	9.73 (6.68)	7.53 (5.81)	< 0.001^b^
Loneliness, mean (SD)	0.53 (0.94)	0.58 (0.97)	0.63 (1.00)	0.44 (0.87)	< 0.001^b^

*Ind. Edu.* individual education, *Neighbor. Edu.* neighborhood education, *HAI* healthy aging index

^a^*P*-value from χ^2^ test

^b^*P*-value from one-way analysis of variance

The mean (SD) years of individual and neighborhood education were 4.89 (4.15) and 4.81 (1.88), respectively, at baseline. Among them, 27.70% and 25.38% had individual education equal to or lower than the neighborhood level. Participants residing in a higher-education neighborhood tended to report better performance and a slow downward trend in HAI score (Supplementary Figures C1 and C2). However, HAI score and its temporal variability differed substantially across combinations of neighborhood and individual education (Supplementary Figures C3 and C4).

### The separate and joint association of individual and neighborhood education with individual-level HAI

**Fig. 3:**
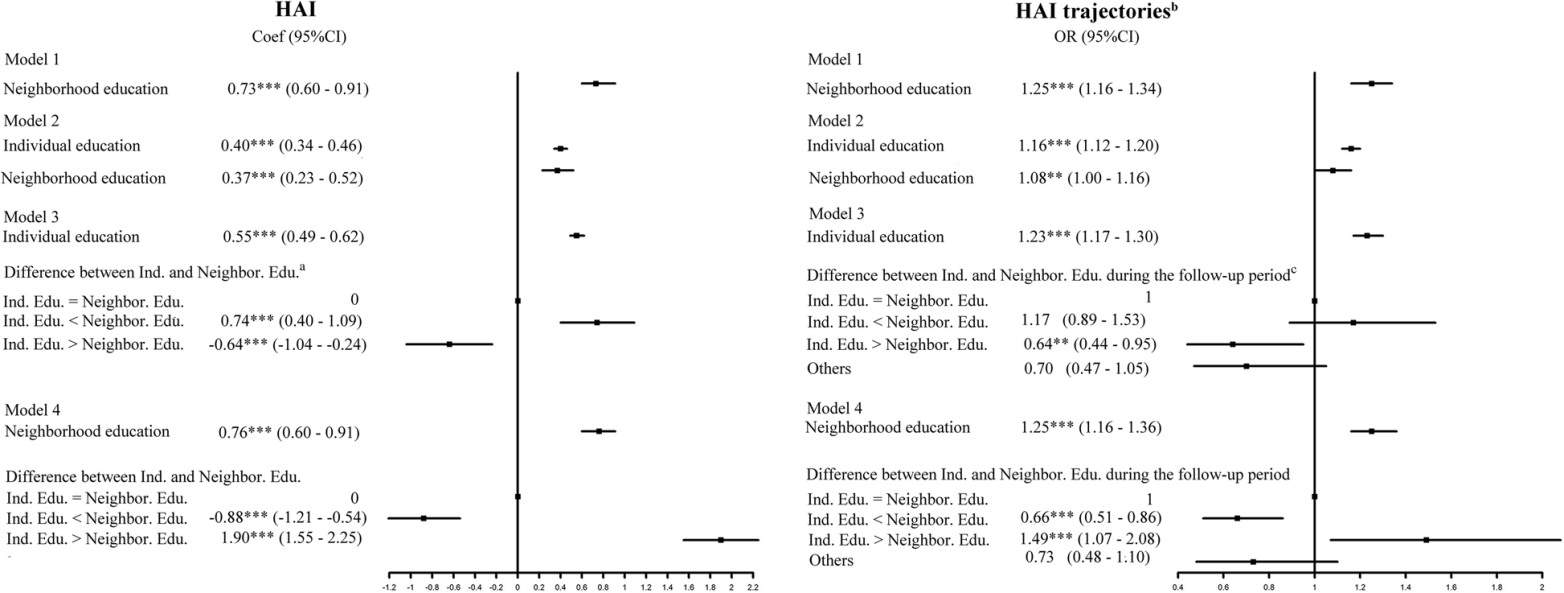
Association between neighborhood education with HAI and HAI trajectory, 2011–2018. *Notes:* Ind. Edu., individual education; Neighbor. Edu., neighborhood education; HAI, healthy aging index. Models were adjusted for age, gender, marital status, area of residence, occupation and frequency of drinking. ^a^Difference between Ind. and Neighbor. Edu.: individual education equal to, lower, or higher than the neighborhood’s at the time of investigation. Association with HAI levels includes 43978 observations for each effect size. ^b^HAI trajectories were grouped as low starting point and fast decline; median starting point and slow decline; high starting point and slow decline; and high starting point with increase followed by decline. The worst health condition group was regarded as reference group. Includes 11,633 observations for each effect size. ^c^Difference between Ind. and Neighbor. Edu. during the follow-up period: individual education equal to, lower, or higher than the neighborhood’s for more than half of the time during follow-up. *p < 0.1, **p < 0.05, ***p < 0.01

Figure 3 (column 1) shows that individual and neighborhood education levels were both significantly and positively associated with individual-level HAI (models 1 and 2). A one-year increase in neighborhood education was independently associated with a 0.37-point increase (95% CI, 0.23–0.52) in individual HAI (model 2), independent of individual education. Model 3 suggested that all individuals, regardless of education, obtained benefits from living in higher-education neighborhoods. Individual-level HAI score grew higher when individuals moved to higher-education neighborhoods. However, the health benefits derived from a neighborhood differed by relative education between individual and neighborhood. The effects of improved neighborhood became weaker (Coef., − 0.88; 95% CI, − 1.21– − 0.54) as neighborhood education increased to a level higher than individual education (model 4). The effect sizes of neighborhood education on each dimension of individual HAI were further estimated. The results were consistent with the overall score, but physical health and functional health were not significantly associated with neighborhood education after controlling for individual education (Supplementary Table C1).

### The separate and joint association of individual and neighborhood education with individual HAI trajectories

Figure 3 (column 2) presents the associations with individual HAI trajectories, which were consistent with those between neighborhood education and individual HAI level. For participants, moving to neighborhoods with a lower average education relative to individual education was detrimental to their HAI trajectory (OR, 0.64; 95% CI, 0.44–0.95) (model 3). Nevertheless, as neighborhood education grew higher than individual education, the effects of improved neighborhood education became weaker with an effect size of 0.66 (95% CI, 0.51–0.86) (model 4).

Supplementary Table C2 shows the descriptive statistics between non-migrated and post-migration observations, which indicated that migrant participants tended to have a higher education levels, live in a more advantaged neighborhoods, and report a higher HAI. Supplementary Tables C3 and C4 show the results using a subsample of residents who had lived in a neighborhood for more than 15 years and age-stratified results. Additionally, regression results with and without imputed data and age-stratified analysis were also consistent (Supplementary Tables C5–C8). There were some numerical discrepancies in the effect sizes for certain estimates, but the main findings remained unchanged.

## Discussion

### Healthy aging

Using nationally representative cohort data in China, this study extracted 16 health indicators and considered interlinkages between indicators to construct an individual HAI score. Consistent with previous literature [5, 11, 14], the mean HAI showed a downward trend with aging, but it seemed that the changes in the HAI were greater than those in previous studies. This divergence may be due to the failure of previous studies to consider health interlinkages [14]. In line with other studies [16], for most participants, worsening healthy aging status was attributed to physical and functional health. Meanwhile, mental health could be improved for some participants, suggesting that prevention and even reversal of aging could occur to some extent.

### The person–neighborhood education fit in healthy aging

Consistent with previous studies, which assessed the effects of neighborhood SES on health [18–20, 30], neighborhood education was found to be independently and positively associated with individual HAI. Furthermore, for the first time, this study found that neighborhood education could contribute to diverging trajectories of HAI. In other words, neighborhood education was associated with widening of the HAI disparities over time, which makes community-based preventative interventions particularly important. In line with previous literature [20, 21, 33], the study found that these health benefits or risks derived from neighborhood resources differed with individual education levels. Moreover, our study contributed to current literature by revealing HAI differences with varying relative education levels between individual and neighborhood. The study found that those benefits from neighborhood were weaker for individuals with lower education relative to the neighborhood average.

The estimated association between neighborhood education and health in our study was consistent with the double jeopardy hypothesis, the fundamental cause theory, and the collective resources model. As predicted by the collective resources model, the study found living in an advantaged neighborhood was always better for health. Furthermore, the double sources of disadvantage reported the lowest HAI, which supports the double jeopardy hypothesis. Having fewer individual and neighborhood resources is expected to take more of a toll on health than if only one type of socioeconomic disadvantage is present. Additionally, fundamental cause theory proposes that individuals with higher SES are more likely to live in neighborhoods with higher SES due to better access to health-enhancing resources [20]. In our sample, migrant participants tended to have higher education levels, live in more advantaged neighborhoods, and report higher HAI. Nevertheless, a subsample analysis among participants who had lived in the same neighborhoods for 15 years revealed that neighborhood education was associated with increased individual HAI.

Contrary to the above three theories and our results, the relative deprivation hypothesis posits that individuals with low SES may be worse off living in neighborhoods with higher SES compared to their counterparts in neighborhoods with lower SES due to the psychological stress of feeling “one-down” [20]. In that theory, others compare themselves to those with similar individual education. People in the same neighborhood could also be regarded as a relevant comparison group due to the frequent interaction within a neighborhood. As individual education was lower than the neighborhood average, they may also have the additional stress of feeling “one-down” and develop a sense of poor person–environment fit. This could explain the reduced benefits from neighborhoods found in our study for those with lower education relative to their neighborhood. Poor person–environment fit not only may cause psychological stress but also may present as lack of ability to make full use of the available resources. An improved neighborhood may entitle residents with lower education levels to more resources, but these people may encounter difficulty in mastering the resources within their environments.

### Policy implications

For the first time, this study constructed an individual HAI accounting for health indicator interlinkages and revealed the person–neighborhood education fit in healthy aging. Although HAI continued to decrease with age, some health indicators, such as life satisfaction, improved among some participants in our study period. Thus, the aging process could be delayed and even reversed. Based on the results in our study, community-level interventions could prompt healthy aging. However, in the neighborhood-improvement process, residents with a lower education level relative to the neighborhood average had poor person–environment fit, leading to health disparities within a neighborhood. Thus, they should be targeted in individually based interventions. Specific to measures, on the one hand, we may seek a way to reduce stress resulting from SES inequality. On the other hand, we may want to ensure each resident has capacity to utilize neighborhood resources. As in a Chinese saying, it is better to teach a man to fish than to give him fish.

### Contribution and limitations

This study extends the person–environment fit in healthy aging including the effects of relative education between individual and neighborhood education. For the first time, the study documents that even those with a high individual education level living in an advantaged neighborhood may encounter poor person–environment fit if their individual education is lower than the neighborhood average. By accounting for relative education between individual and neighborhood, the competing theoretical models on the joint association of individual and neighborhood education could be coordinated. Second, the proposed hybrid method enabled us to overcome the shortcomings of previous studies in making a comprehensive assessment of healthy aging by considering these identified interrelations between health indicators.

The study has several limitations. First, the study is limited by sample selection. On the one hand, fundamental cause theory suggests those with high education levels may choose to live in advantaged neighborhoods [20]. On the other hand, some participants in the first or second survey died and therefore did not reply to the subsequent surveys [14]. As such, cohort weight was applied to reduce the bias. In addition, the study used subsample analysis for long-term residents to deal with the migration bias. An extra source of bias can be that health measurements were retrospective self-evaluations [34].

Second, the study only used neighborhood education to test the person–environment fit in healthy aging. SES is multidimensional, and education is just one dimension [16]. Since education is not affected by health at an older age [21], we used education as a proxy for SES to avoid the potential endogeneity issue caused by the reversed association between current health and some SES indicators (e.g., income, employment, and wealth) in the corresponding period. However, the results from the study should be applied to other dimensions of SES with caution. More sophisticated approaches, such as instrumental variables approach, should be used to correctly capture the effects of relative SES between individual and neighborhood.

Third, the study is only an observation study. At this stage, no empirical work has been done to verify the hypotheses or theories. Therefore, the underlying mechanisms through which neighborhood SES operates on individual healthy aging remain unknown and require further investigation in future studies.

Fourth, the relationship between education level and these 16 indicators is so complex that the association of education and HAI cannot cover all the effects of neighborhood education on different health indicators. Nevertheless, factor analysis results in a reduction of the multidimensional parameter space into the HAI with four dimensions, which accounted for 60% of the total variance. Thus, this study may not be generalizable to other countries. Nevertheless, the study highlights the importance of person–environment fit in healthy aging, which may have universal value.

## Conclusion

Using national cohort data from 2011 to 2018, the study extracted 16 health indicators to construct an individual HAI score accounting for health indicator interactions. Neighborhood education was found to be independently associated with improved HAI at the individual level. However, individuals with lower education relative to the neighborhood average tended to benefit less from neighborhood resources due to poor person–environment fit. Thus, to create more age-friendly environments, community-based interventions should be combined with individual-based interventions. The individual-based interventions targeting individuals with lower relative education aim to address poor person–environment fit in the process of neighborhood promotion.

## Supplementary Information

**Additional file 1.** Supplementary materials.

## Data Availability

The datasets used and analyzed during the current study are available in http://charls.pku.edu.cn/en/.

## Abbreviations

HAI: Healthy aging index
CHARLS: China Health and Retirement Longitudinal Study
WHO: World Health Organization
SES: Socioeconomic status
MI: Multiple imputation
ADLs: Activities of daily living
IADLs: Instrumental activities of daily living
TOPSIS: Technique for order preference by similarity to an ideal solution
CIs: Confidence intervals
GBTM: Group-based trajectory modeling
ORs: Odds ratios
