# Supplementary material for: Healthy aging index and its link with relative education between individual and neighborhood: a population-based, cohort study
Source: BMC Geriatr. 2022 Oct 3;22:778. doi: 10.1186/s12877-022-03469-7 (PMC9528066; doi:10.1186/s12877-022-03469-7)
Supplement: Supplementary file 1 — Additional file 1. Supplementary materials. [file 12877_2022_3469_MOESM1_ESM.docx]

**Supplementary materials**

**Supplementary Text 1.** Multiple imputation

**Supplementary Text 2.** The construction of HAI

**Supplementary Text 3.** Analytic results of HAI

**Supplementary Text 4.** Group-based trajectory modeling

**Supplementary Table C1**. Association between neighborhood education and dimensions of HAI with imputed data

**Supplementary Table C2**. Descriptive statistics by migration

**Supplementary Table C3.** Association between neighborhood education and HAI levels for participants residing in the same neighborhood for 15 years with imputed data

**Supplementary Table C4.** Association between neighborhood education and HAI trajectories for participants residing in the same neighborhood for 15 years with imputed data

**Supplementary Table C5.** Age stratified association between neighborhood education and HAI levels with imputed data

**Supplementary Table C6.** Age stratified association between neighborhood education and HAI trajectories with imputed data

**Supplementary Table C7.** Association between neighborhood education and HAI levels for complete cases

**Supplementary Table C8.** Association between neighborhood education and HAI trajectories for complete cases

**Supplementary Figure C1.** HAI levels by neighborhood education

**Supplementary Figure C2.** HAI trajectories by neighborhood education

**Supplementary Figure C3**. HAI levels by neighborhood education and individual education

**Supplementary Figure C4**. HAI trajectories by neighborhood education and individual education

**Supplementary References**

**Supplementary Text 1. Multiple imputation**

Multiple imputation (MI) with chained equations was used to impute any missing data by creating 10 imputed datasets. There were 9855 observations excluded because of missing value (at a 22.4% missing rate). Details of the names and rates of variables with missing data are presented in flow chart (Figure 1). The missing values occurred mostly for health indicators, such as diagnosis with any chronic disease, and mobility measurements; there were no data missing for age, gender, residence, marital status, and education. Differences between individuals with complete and incomplete data are presented in Table 1a of the Supplementary Text. Those with missing values were older, and more were women than men. Missing health measurements were worse than measured health but only because older people may be more likely to have missing health indicators. We thus assume the missing data are Missing At Random (MAR), especially given the large sample size. Hence, multiple imputation (MI) with chained equations was applied to impute any missing data. MI was performed using the R multivariate imputation by chained equation package [1].

The MI process was conditional on age, gender, residence, marital status, and education. Age was included in the imputation procedure, making MAR plausible. Log and logit transformations were used to address non-normality (e.g., occupation, whether diagnosed with hypertension by a doctor, long distance vision functions, and life satisfaction) in the MI process. Meanwhile, continuous variables (e.g., cognitive functions, number of outpatient and inpatient visits) were dealt with linear regression. Complete data were transformed back to their original scales before analysis. Ten imputed datasets were created. Density plots were provided for visual representations of the extent to which imputed values differ from observed values. According to the distribution of complete and imputed data, the set of imputed data closest to the complete data distribution was selected.

**Supplementary Text 1a. Descriptive statistics by incomplete and complete data**

| **Characteristics** | No. (%) |  | *p* value |
| --- | --- | --- | --- |
|  | Incomplete data | Complete data |  |
|  | n=9855 | n=34123 |  |
| **Women** | 5709 (57.93) | 17548 (51.43) | <0.001^a^ |
| **Age, mean (SD)** | 63.71 (10.92) | 60.83 (8.72) | <0.001^a^ |
| **Urban area of residence** | 2836 (28.78) | 10603 (31.07) | <0.001^a^ |
| **Individual education, mean (SD)** | 3.70 (3.93) | 5.11 (4.11) | <0.001^b^ |
| **Neighborhood education, mean (SD)** | 4.66 (1.82) | 4.96 (1.93) | <0.001^b^ |
| **Occupation** |  |  | <0.001^a^ |
| Unemployed | 3720 (38.45) | 10323 (30.25) |  |
| Agricultural workers | 4563 (47.17) | 18125 (53.12) |  |
| Self-employed | 577 (5.96) | 2602 (7.63) |  |
| Managers and professionals | 814 (8.41) | 3073 (9.01) |  |
| **Unmarried** | 2311 (23.45) | 6147 (18.01) | <0.001^a^ |
| **Frequency of drinking** |  |  | 0.001^a^ |
| Drink more than once a month | 2058 (21.23) | 9217 (27.01) |  |
| Drink but less than once a month | 724 (7.47) | 2593 (7.60) |  |
| None | 6912 (71.30) | 22313 (65.39) |  |
| **Health indicators** |  |  |  |
| Cognitive functions, mean (SD) | 6.90 (6.69) | 13.32 (5.65) | <0.001^b^ |
| Social participations, mean (SD) | 0.58 (0.87) | 0.87 (1.07) | <0.001^b^ |
| Number of outpatient visits, mean (SD) | 0.40 (1.39) | 0.37 (1.36) | 0.062^b^ |
| Number of hospitalizations, mean (SD) | 0.17 (0.65) | 0.15 (0.58) | 0.002^b^ |
| Number of chronic diseases, mean (SD) | 2.04 (1.73) | 1.97 (1.67) | <0.001^b^ |
| Self-reported health, mean (SD) | 3.16 (1.01) | 3.09 (1.01) | <0.001^b^ |
| Mobility, mean (SD) | 14.83 (6.20) | 12.71 (4.46) | <0.001^b^ |
| ADLs, mean (SD) | 7.03 (2.57) | 6.41 (1.27) | <0.001^b^ |
| IADLs, mean (SD) | 6.91 (3.71) | 5.70 (1.89) | <0.001^b^ |
| Number of disabilities, mean (SD) | 0.64 (0.93) | 0.39 (0.70) | <0.001^b^ |
| Lone distance vision functions, mean (SD) | 3.91 (0.98) | 3.73 (0.99) | <0.001^b^ |
| Near vision functions, mean (SD) | 3.89 (0.95) | 3.78 (0.95) | <0.001^b^ |
| Hearing functions, mean (SD) | 3.76 (0.96) | 3.58 (0.94) | <0.001^b^ |
| [Life](javascript:;) [satisfaction](javascript:;), mean (SD) | 2.86 (0.84) | 2.78 (0.75) | <0.001^b^ |
| Depressive symptoms, mean (SD) | 9.37 (6.61) | 8.21 (6.25) | <0.001^b^ |
| Loneliness, mean (SD) | 0.66 (1.07) | 0.50 (0.94) | <0.001^b^ |

*Notes:* ^a^ P-value from χ^2^ test.

^b^ P-value from one-way analysis of variance.

**Supplementary Text 2. The construction of healthy aging index**

The study proposes a hybrid method, that is, integrating network analysis with technique for order preference by similarity to an ideal solution (TOPSIS) approach, to construct healthy aging index (HAI) accounting for health indicators interlinkages. The specific process was as follows:

**Step 1 Data collection**

According to WHO, health is “a state of complete physical, mental and social well-being and not merely the absence of disease or infirmity”, and healthy aging is “the process of developing and maintaining the functional ability that enables wellbeing in older age [2,3]” Hence, four dimensions were discussed while assessing healthy aging: functional abilities, intrinsic capacities, well-being, and environments. The first three dimensions were included as indicator pool of HAI, and the relationship between environments characteristics and the HAI were estimated through regression models. Our fixed personal characteristics (e.g., gender, marital status), social norms (e.g., occupation, education), and other factors (e.g., drinking) across our life span can affect health outcomes, which were adjusted for in the regress analysis. Functional abilities refer to health-related attributes that allow people to do what they have reason to value; thus, the indicators cover activities of daily living [ADLs], instrumental ADLs [IADLs], mobility, disabilities, hearing and sighting functions in our study [3–8]. Intrinsic capacities suggest all the physical and mental capabilities that an individual can draw on [3,4,9]. Accordingly, presence of chronic diseases, inpatient and outpatient visits were proposed to measure physical capabilities, while depression symptoms, loneliness, and life satisfaction for mental capabilities [3,9,10]. Well-being refers to happiness, security, and fulfilment, measured by life satisfaction, cognitive functions, and social participations [3,5,6]. According to a review, many studies considered individuals’ general health status while assessing healthy aging, and thus self-rated health was included in our study [4]. Using data from CHARLS 2011, 2013, 2015 and 2018, the above 16 indicators comprised the health indicator pool.

**Supplementary Text 2a. Health indicators questionnaires**

| **Dimensions** | **Health indicators** | **Measurement of indicators** | **Coding of variables** |
| --- | --- | --- | --- |
| Well-being | Cognitive functions | Cognitive functions were assessed by two measurements, episodic memory and mental intactness. Episodic memory was assessed through word recall test (immediate recall and delayed recall). Mental intactness consisted of numerical ability, time orientation and picture drawing. | The cognitive functions were the summation of each part. Total scores ranged from 0 to 30.  Higher scores represented higher cognitive functions. |
|  |  |  |  |
|  | Social participations | Participants were asked whether participating in social activities in the month preceding the interview, including “voluntary or charity work”, “provided help to family, friends or neighbors”, “gone to a sport, social or other kind of club”, “played Mahjong, chess, cards, or went to community club”, “took part in a community-related organization”, “cared for a sick or disabled adult who does not live with you”, “attended an educational or training course”, “stock investment”, “used the Internet”, “others”. | Yes=1; no=0  The answers to the 11 questions were added up to get the final score, ranging from 0 to 11. |
|  |  |  |  |
| Physical and functional health | Number of chronic diseases | Participants were asked whether a doctor had told them that they had any of the following major chronic diseases: Hypertension; Dyslipidemia; Diabetes or high blood sugar; Cancer or malignant tumor; Chronic lung diseases, such as chronic bronchitis , emphysema; Liver disease; Heart attack, coronary heart disease, angina, congestive heart failure, or other heart problems; Stroke; Kidney disease; Stomach or other digestive disease (except for tumor or cancer); Emotional, nervous, or psychiatric problems; Memory-related disease; Arthritis or rheumatism; Asthma. | Yes=1; no=0  The answers to the 14 chronic diseases were added up to get the final score, ranging from 0 to 14. |
|  | Mobility | Measured body function limitations. Participants were asked whether having difficulty in performing the following tasks on a regular basis: running or jogging about 1 Km; walking 1 km; walking 100m; getting up from a chair; climbing several flights of stairs; stooping, kneeling or crouching; reaching or extending your arms above shoulder level; lifting or carrying items weighing 5 kg; and picking up a small coin from a table. | “Completing the task with not having any difficulty” received 1 point, “having difficulty but can still do it” received 2 points, “having difficulty and needing help” received 3 points, “not doing it” received 4 points. The scores of the 9 items were summed with a maximum of 36 points and a minimum of 9 points.  The higher scores expressed worse health condition. |
|  | ADLs | Measured the abilities of self-care. Participants were asked whether having difficulty in dressing, bathing, eating, getting into or out of bed, toileting. | The response was the same as mobility. The scores of the 6 items were summed with a maximum of 24 points and a minimum of 6 points. |
|  |  |  |  |
|  | IADLs | Participants were asked whether having difficulty in doing household chores, preparing hot meals, shopping for groceries, taking medications and managing money. | The response was the same as mobility. The scores for the 5 items were summed with a minimum of 5 points and a maximum of 20 points. |
|  |  |  |  |
|  | Number of disabilities | Included physical disabilities, brain damage, vision problem, hearing problem and speech impediment | Yes=1; no=0  The answers to the 5 disabilities condition were added up to get the final score, ranging from 0 to 5 |
|  |  |  |  |
| Sensory health | Long distance vision functions | Participants were asked, “How good is your eyesight for seeing things at a distance”. | Values of 1-5 represented: excellent, very good, good, fair, poor. |
|  |  |  |  |
|  | Near vision functions | Participants were asked, “How good is your eyesight for seeing things up close”. | Values of 1-5 represented: excellent, very good, good, fair, poor. |
|  |  |  |  |
|  | Hearing functions | Participants were asked, “Is your hearing very good, good, fair, poor, or very poor” | values of 1-5 represented: excellent, very good, good, fair, poor. |
|  |  |  |  |
| Mental health | Life satisfaction | “How satisfied with life-as-a-whole” | Values of 1-5 represented: completely satisfied, very satisfied, somewhat satisfied, not very satisfied, not at all satisfied. |
|  |  |  |  |
|  | Depressive symptoms | The 10-item Center for Epidemiologic Studies Depression Scale (CES-D10) was used to assess depressive symptom, which has been identified as a valid, reliable, and useful mental health assessment tool for older people in China. This scale included two positive items and eight negative items. | The CES-D 10 included 10 items with 4 answers on a 4-scale metric, ranging from rarely or none of the time (<1 day), some days (1-2 days), occasionally (3-4 days), or most of the time (5-7 days). Negative items were scored from 0 to 3, positive items were scored from 3 to 0, with a total possible score ranging from 0 to 30.  A higher score reflected more severe depressive symptom. |
|  |  |  |  |
|  | Loneliness | In the last week, how often did you feel lonely? | The item had 4 answers on a 4-scale metric, ranging from rarely or none of the time (<1 day), some days (1-2 days), occasionally (3-4 days), or most of the time (5-7 days), scored from 0 to 3. |
|  |  |  |  |
|  | Number of outpatient visits | How many times did you visit a general hospital, specialized hospital, clinic, or other medical facilities for outpatient care in the past month? |  |
|  |  |  |  |
|  | Number of hospitalizations | How many times did you stay in hospital over the past year? |  |
|  |  |  |  |
|  | Self-reported health | “Would you say your health is very good, good, fair, poor or very poor?” | Values of 1-5 represented: excellent, very good, good, fair, poor. |

*Notes:* ADLs, activities of daily living; IADLs, instrumental ADLs

**Step 2 Normalization health indicators**

The collected health indicators covered diverse units and value range, increasing the complexity of identifying the interdependent relationships and structuring health indicators. Hence, all health indicators should be positively normalized before screening [11]. Given our data characteristics (the existence of zero in the raw statistics), the maximum and minimum method was used to normalize these health indicators. Suppose that $a_{ij}^{t}$ was the original data value of indicator, $i=1, 2, 3,\cdots,16$ meant the types of health indicators, and $j=1, 2, 3,\cdots, 11633$represented the participants under different years $t=2011, 2013, 2015, 2018$. For the positive indicators, which were the cognitive functions and social participations, the normalization procedure needed to apply the following equation:

$a_{ij}^{t+}=\frac{a_{ij}^{t}-a_{i}^{min}}{a_{i}^{max}-a_{i}^{min}}, a_{ij}^{t+}\in[0,1]$ (1)

For the negative indicators (e.g., the other 14 health indicators), the normalization procedure needed to apply the following equation:

$a_{ij}^{t-}=\frac{a_{i}^{max}-a_{ij}^{t}}{a_{i}^{max}-a_{i}^{min}}, a_{ij}^{t-}\in[0,1]$ (2)

Where $a_{i}^{max}$ and $a_{i}^{min}$ represented the best and worst value for health indicators over all observations from 2011 to 2018. All normalized values were multiplied by 100, ranged from 0 to 100.

**Step 3 Dimensions of HAI**

Once these health indicators were normalized into unit-free values, exploratory factor analysis (EFA) was used to extract the effects to structure different dimensions of HAI by gathering their factor loadings. The total sample was randomly divided into two halves using random number method. EFA was performed using a goemin oblique rotation in the first sample. The second sample was used to perform confirmatory factor analysis (CFA) for validating the EFA structure [12-14]. The model fit test was evaluated using the Tucker-Lewis index (TLI), the comparative fit index (CFI) and the root mean square error of approximation (RMSEA). For the TLI and CFI, values between 0.90 and 0.95 were considered acceptable, and 0.95 as good in EFA and CFA. RMSEA values less than 0.05 indicates good fit. Factor analyses were performed using the Mplus program.

In order to further confirm the dimensions of HAI, hierarchical cluster analysis (HCA) was conducted to draw a whole picture of how health indicators tend to cluster together [15]. It was used to identify homogeneous dimensions from the dissimilarities between health indicators by creating a cluster tree, or dendrogram [16]. To perform agglomerative HCA, first the dissimilarity between every pair of all health indicators was measured by Euclidean distance. Second, health indicators were paired into binary clusters based on distance information. The smaller the distance, the higher the similarity. Then newly formed clusters were grouped into larger clusters until the dendrogram was formed.

**Step 4 Network analysis**

Following CFA, EFA and HCA, network analysis was applied to understand the relative importance of health indicators in HAI through health indicators interlinkages [17,18]. Firstly, an adjacency matrix for evaluating the correlations was examined across different health indicators. Spearman’s correlation was conducted to acquire the monotonic relations among all the possible combinations for the health indicators. Afterward, based on the adjacency matrix, the interlinkages between indicators for presenting the contribution of each health indicator under the healthy aging network was calculated using the eigenvector centrality. The eigenvector centrality supposes that the influence of a node was not only determined by the number of its neighbors, but also determined by the influence of each neighbor. The following equation can be employed to identify the eigenvector centrality for each health indicator.

$x_{k}=\lambda^{-1}\sum_{k=1}^{i} m_{i}e_{i}$ (3)

where $m_{i}$ was the adjacency matrix, which was determined from Spearman’s correlation; $\lambda$ and $e_{i}$ were the largest eigenvalue and the corresponding eigenvector for the $m_{i}$.

**Step 5 Calculating the HAI through TOPSIS**

According to the contribution of indicators $x_{k}$ as weights, the comprehensive HAI was calculated by TOPSIS [19,20]. The procedure was as follows:

**(1) Construct the normalized decision matrix**

The normalizing data were arranged into a decision-making matrix, as shown in the following equation.

$D^{t}=\left[ \begin{matrix} \begin{matrix} a_{11}^{t} & a_{21}^{t} \end{matrix} & \cdots& a_{m1}^{t} \\ \begin{matrix} a_{12}^{t} & a_{22}^{t} \end{matrix} & \cdots& a_{m2}^{t} \\ \begin{matrix} \begin{matrix} \vdots\\ a_{1n}^{t} \end{matrix} & \begin{matrix} \vdots\\ a_{2n}^{t} \end{matrix} \end{matrix} & \begin{matrix} \ddots\\ \cdots\end{matrix} & \begin{matrix} \vdots\\ a_{mn}^{t} \end{matrix} \end{matrix} \right]_{i\times j} , m=1,2,\cdots, i; n=1,2,\cdots, j$ (4)

**(2) Determine each indicator’s positive and negative ideal solutions**

Positive ideal solution:

$I^{tp}=\left( a_{1n}^{t+}, a_{2n}^{t+}, \cdots, a_{mn}^{t+} \right)=\left( {}_{1\leq n\leq j}^{max}{a_{1n}^{t}},{}_{1\leq n\leq j}^{max}{a_{2n}^{t}},\cdots, {}_{1\leq n\leq j}^{max}{a_{mn}^{t}} \right)$ (5)

Negative ideal solution:

$I^{tn}=\left( a_{1n}^{t-}, a_{2n}^{t-}, \cdots, a_{mn}^{t-} \right)=\left( {}_{1\leq n\leq j}^{min}{a_{1n}^{t}},{}_{1\leq n\leq j}^{min}{a_{2n}^{t}},\cdots, {}_{1\leq n\leq j}^{min}{a_{mn}^{t}} \right)$ (6)

**(3) Measure the Euclidean distance from each target value to positive and negative ideal solutions of each indicator**

$\theta_{n}^{tp}=\sqrt{\sum_{k=1}^{i} {x_{k}\left( a_{mn}^{t+}-a_{ij}^{t} \right)}^{2}}$ (7)

$\theta_{n}^{tn}=\sqrt{\sum_{k=1}^{i} x_{k}\left( a_{mn}^{t-}-a_{ij}^{t} \right)^{2}}$ (8)

**(4) Calculate the relative closeness to the ideal solution of each observation**$\omega_{n}^{t}$

$\frac{\theta_{n}^{tn}}{\theta_{n}^{tp}-\theta_{n}^{tn}}, \omega_{n}^{t}\in\left[ 0,1 \right]$ (9)

Based on these weights, this study used 13 health indicators to identify each observation’s HAI.

**Supplementary Text 3. Analytic results of HAI**

The results of the construction of HAI are shown in table 1. The selected 16 health indicators from CHARLS were described as follows. The mean (SD) of cognitive functions was 12.04 (6.31), while for social participations, the mean (SD) was 0.71 (0.90), with the mean (SD) of depressive symptoms and life satisfaction score being 8.48 (6.26) and 2.95 (0.71), respectively. Older people often suffered from at least one chronic disease (mean [SD] = 1.36 [1.39]), and number of outpatient visits and hospitalizations (SD) were 0.41 (1.41) and 0.14 (0.59). The score of long distance vision functions and near vision functions (SD) were 3.69 (0.99) and 3.80 (0.93), with ADLs and IADLs being 6.41 (1.39) and 5.73 (2.01).

Indicators with factor loading greater than 0.3 were selected out and divided into one dimension, the result of dimension structure are shown as follows. Dimension 1 included cognitive functions and social participations, defined as well-being [3]. Dimension 2 included mobility, ADLs, IADLs, number of disabilities and chronic diseases, representing physical and functional health [3]. Dimension 3 was sensory health including long distance vision functions, near vision functions and hearing functions [7]. Dimension 4 included life satisfaction, depressive symptoms and loneliness, which indicated mental health [7]. The model fitting parameters CFI and TLI were 0.96, 0.90 in EFA, 0.93, 0.87 in CFA, the value of RMSEA was 0.044 in our model. Judging through the above three parameters, the CFA and EFA model were considered acceptable.

**Supplementary Text 3a. Dimension in HAI**

| **Pattern Matrix** | **EFA** | | | | **CFA** | | | |
| --- | --- | --- | --- | --- | --- | --- | --- | --- |
| Item code | **Factor1** | **Factor2** | **Factor3** | **Factor4** | **Factor1** | **Factor2** | **Factor3** | **Factor4** |
| Cognitive functions | **0.85** | 0.01 | -0.01 | 0.02 | 0.72 |  |  |  |
| Social participations | **0.30** | 0.02 | 0.07 | 0.03 | 0.39 |  |  |  |
| Number of outpatient visits | -0.01 | 0.01 | 0.03 | 0.03 |  |  |  |  |
| Number of hospitalizations | 0.01 | 0.07 | 0.02 | 0.02 |  |  |  |  |
| Number of chronic diseases | -0.14 | **0.30** | 0.18 | 0.13 |  | 0.38 |  |  |
| Self-reported health | -0.03 | 0.11 | 0.15 | 0.09 |  |  |  |  |
| Mobility | 0.05 | **0.71** | 0.10 | 0.07 |  | 0.82 |  |  |
| ADLs | -0.07 | **0.86** | -0.06 | -0.02 |  | 0.78 |  |  |
| IADLs | 0.05 | **0.80** | -0.02 | -0.03 |  | 0.80 |  |  |
| Number of disabilities | 0.11 | **0.37** | 0.17 | -0.01 |  | 0.44 |  |  |
| Long distance vision functions | 0.02 | 0.01 | **0.71** | -0.01 |  |  | 0.73 |  |
| Near vision functions | -0.03 | -0.03 | **0.62** | -0.01 |  |  | 0.59 |  |
| Hearing functions | 0.05 | 0.03 | **0.56** | 0.01 |  |  | 0.55 |  |
| [Life](javascript:;) [satisfaction](javascript:;) | -0.04 | -0.04 | 0.06 | **0.38** |  |  |  | 0.34 |
| Depressive symptoms | 0.02 | 0.01 | 0.01 | **0.98** |  |  |  | 1.01 |
| Loneliness | 0.02 | 0.02 | -0.05 | **0.64** |  |  |  | 0.60 |

*Notes:* HAI, healthy aging index; EFA, exploratory factor analysis; CFA, confirmatory factor analysis; ADLs, activities of daily living; IADLs, instrumental ADLs.

To further confirm the decomposition of different dimensions of HAI, this study used HCA to draw a whole picture of how health indicators tend to cluster together. From the dendrograms resulting from clusters analysis showing as follows, the physical and functional health cluster (ADLs, IADLs, mobility, number of disabilities and chronic diseases) formed a cluster grouped into larger cluster with mental health cluster (depressive symptoms, life satisfaction and loneliness). These two clusters were grouped into sensory health cluster (long distance vision functions, near vision functions and hearing functions), finally combined with well-being cluster (cognitive functions and social participations) until the dendrogram was formed. The results were consistent with factor analysis for the classification of health aging dimensions.


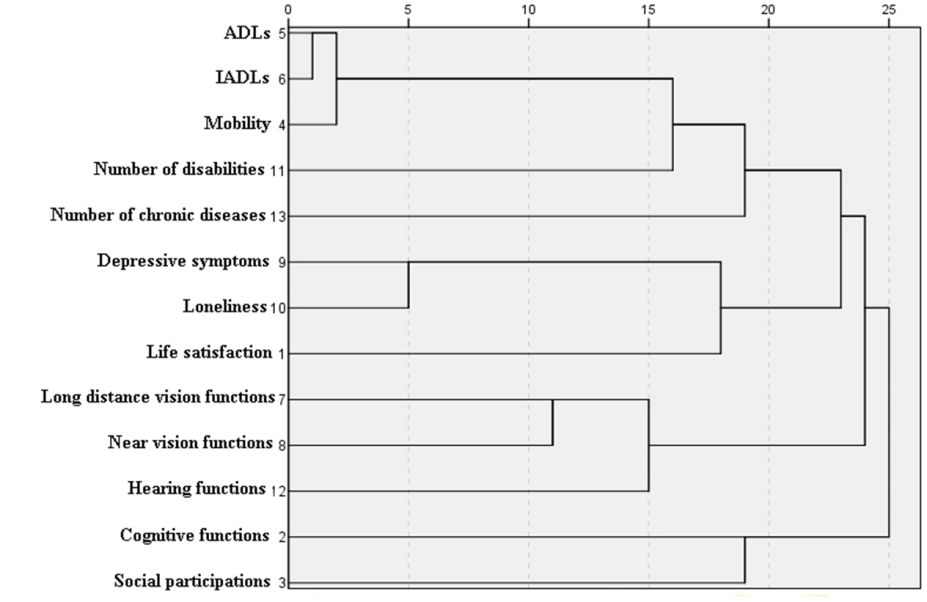


**Supplementary Text 3b. Cluster groups of HAI from HCA**

*Notes:* ADLs, activities of daily living; IADLs, instrumental ADLs; HAI, healthy aging index; HCA, hierarchical cluster analysis.

Subsequently, Eq. (3) was applied to obtain the eigenvector centrality to clarify the contributions for each health indicator to HAI. According to the result of network analysis, mobility was the most important indicator in measuring HAI, with a weight of 0.400, followed by ADLs (0.338), IADLs (0.351), number of disabilities (0.280) and number of chronic diseases (0.260) in physical and functional health. For mental health, depressive symptoms and loneliness had higher weight, which were 0.357 and 0.248 respectively, while life satisfaction had a relatively low weight with 0.097. The weight of cognitive functions and social participations were respectively 0.224 and 0.095 in well-being. For sensory health, each of the three indicators’ weights was 0.289 for long distance vision functions, 0.207 for near vision functions, and 0.269 for hearing functions. The following health indicators network diagram shows the relative importance of health indicators in HAI and interlinkages between health indicators.


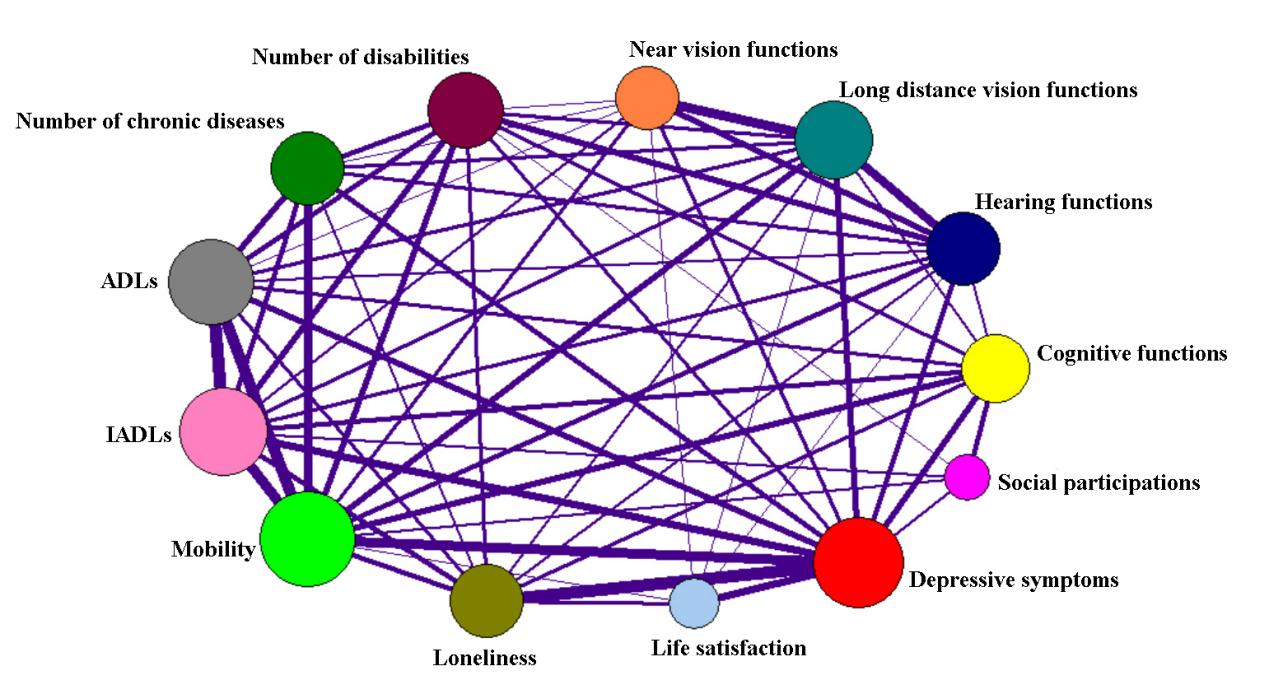


**Supplementary Text 3c.** **Health indicators network diagram**

*Notes:* ADLs, activities of daily living; IADLs, instrumental ADLs.

The thickness of the line represents the correlation degree of health indicators. The thicker the line, the higher the correlation between health indicators.

The circle represents 13 health indicators, and the greater the area of the circle, the greater the weight of indicators.

We obtained the TOPSIS results by employing Eq. (4) - (9). Among the 11633 participants, the mean (SD) HAI was 48.94 (7.55) at baseline, showing a downward trend with age. The higher the neighborhood education, the better the HAI for older people.

**Supplementary Text 4. Group-based trajectory modeling**

Group-based trajectory modeling (GBTM) was applied to identify the development of the HAI across the whole life process at the individual level [21]. Using GBTM, the HAI trajectories for all respondents were classified into four groups: low starting point and fast decline, median starting point and slow decline, high starting point and slow decline, and high starting point with increase followed by decline. These reflect the temporal variation in the HAI from 2011 to 2018.

Maximum likelihood estimation was used to estimate the parameters of the model and identify clusters of individuals with similar trajectories while assuming the concentration data distributed as the censored normal distribution. Analyses were conducted using Traj macro in STATA. The number of trajectory groups that best fit the data was determined using the lower Akaike and Bayesian Information Criteria (AIC and BIC) values; the accuracy of the classification (average posterior probability (AvePP ≥ 0.70)) and the overall adequacy and interpretability of the model (≥5% of participants were assigned to each trajectory grouping) of our participants were also considered. Each participant was assigned to the group for which their average posterior probability was greatest. As shown in the following table, although the three groups had higher entropy than the four groups, the four groups had lower AIC and BIC, and over five-percent of participants were assigned to each trajectory grouping. Thus, the four groups were selected.

Afterward, the shapes of each trajectory were determined in a stepwise manner, starting with the highest polynomial (e.g., cubic function) for each group trajectory. If the highest polynomial was not statistically significantly different from 0 (p > 0.05), we replaced it with a lower polynomial function (e.g., quadratic or linear function) [22]. The HAI trajectories were finally classified into the following forms: low starting point and fast decline, median starting point and slow decline, high starting point and slow decline, and high starting point with an increase followed by decline.

**Supplementary Text 4a. Fit information for the GBTM**

| **Groups** | **AIC** | **BIC** | **Entropy** | **Percent (%)** | **AvePP for each group** |
| --- | --- | --- | --- | --- | --- |
| 2 | -147973.2 | -148006.3 | 0.699 | 45.13%/54.87% | 0.90/0.92 |
| 3 | -145925 | -145972.8 | 0.759 | 17.53%/61.45%/21.02% | 0.88/0.89/0.87 |
| 4 | -145201.3 | -145278.6 | 0.731 | 9.12%/40.42%/42.22%/8.24% | 0.87/0.84/0.83/0.86 |
| 5 | -145017.3 | -145109.3 | 0.714 | 4.99%/23.73%/47.99%/20.39%/2.90% | 0.85/0.79/0.80/0.80/0.85 |

**Supplementary Table C1. Association between neighborhood education and dimensions of HAI with imputed data**

|  | Well-being | Physical and functional health | Sensory health | Mental health |
| --- | --- | --- | --- | --- |
|  | Coef (95% CI) | Coef (95% CI) | Coef (95% CI) | Coef (95% CI) |
| **Model 1^a^** | | | | |
| Neighborhood education | 1.87***  (1.54 - 2.20) | 0.46***  (0.31 - 0.61) | 0.76***  (0.49 - 1.02) | 0.91***  (0.66 - 1.16) |
| **Model 2^a^** | | | | |
| Individual education | 1.19***  (1.07 - 1.30) | 0.30***  (0.25 - 0.36) | 0.34***  (0.23 - 0.46) | 0.39***  (0.28 - 0.49) |
| Neighborhood education | 0.73***  (0.40 - 1.06) | 0.18**  (0.02 - 0.33) | 0.43***  (0.15 - 0.70) | 0.54***  (0.28 - 0.80) |
| **Model 3^a^** | | | | |
| Neighborhood education | 1.88***  (1.54 - 2.21) | 0.47***  (0.32 - 0.62) | 0.75***  (0.48 - 1.02) | 0.91***  (0.66 - 1.15) |
| Difference between Ind. and Neighbor. Edu.^b^ | | | | |
| Ind. Edu. = Neighbor. Edu. | 0 | 0 | 0 | 0 |
| Ind. Edu. < Neighbor. Edu. | -4.52***  (-5.21 - -3.84) | -1.30***  (-1.81 - -0.78) | 0.09  (-0.65 - 0.84) | -0.64  (-1.53 - 0.25) |
| Ind. Edu. > Neighbor. Edu. | 4.82***  (3.93 - 5.72) | 1.36***  (0.94 - 1.77) | 1.95***  (1.17 - 2.73) | 2.18***  (1.38 - 2.99) |
| **Model 4^a^** | | | | |
| Individual education | 1.42***  (1.25 - 1.58) | 0.34***  (0.27 - 0.41) | 0.57***  (0.44 - 0.70) | 0.59***  (0.47 -0.72) |
| Difference between Ind. and Neighbor. Edu. | | | | |
| Ind. Edu. = Neighbor. Edu. | 0 | 0 | 0 | 0 |
| Ind. Edu. < Neighbor. Edu. | -0.13  (-0.72 - 0.44) | -0.17  (-0.68 - 0.33) | 1.64***  (0.83 - 2.44) | 1.17**  (0.25 - 2.09) |
| Ind. Edu. > Neighbor. Edu. | -1.59**  (-2.56 - -0.61) | -0.12  (-0.60 - 0.36) | -0.75*  (-1.65 - 0.14) | -0.32  (-1.38 - 0.74) |

*Notes*: Coef, coefficient; CI, confidence interval; Ind. Edu., individual education; Neighbor. Edu., neighborhood education.

^a^ Models were adjusted for age, gender, marital status, area of residence, occupation and frequency of drinking, including 43978 observations for each effect size.

^b^ Difference between Ind. and Neighbor. Edu.: individual education equal to, lower, or higher than the neighborhood’s at the time of investigation.

*p < 0.1, **p < 0.05, ***p < 0.01.

**Supplementary Table C2. Descriptive statistics by migration**

|  | No. (%) |  |  |
| --- | --- | --- | --- |
| **Characteristics** | Non-migrated observations  (n=43978) | Post-migration observations  (n=2554) | P value |
| **Gender** |  |  |  |
| Men | 20721 (47.12%) | 1230 (48.16%) | 0.305^a^ |
| Women | 23257 (52.88%) | 1324 (51.84%) |  |
| **Age, mean (SD)** | 61.45 (9.31) | 61.41 (8.57) | 0.820^b^ |
| **HAI, mean (SD)** | 48.35 (7.95) | 48.68 (8.43) | 0.050^b^ |
| **Area of residence** |  |  |  |
| Rural | 30539 (69.44%) | 1578 (61.79%) | <0.001^a^ |
| Urban | 13439 (30.56%) | 976 (38.21%) |  |
| **Married** |  |  |  |
| Married | 35520 (80.77%) | 1910 (74.78%) | <0.001^a^ |
| Unmarried | 8458 (19.23%) | 644 (25.22%) |  |
| **Individual education, mean (SD)** | 4.80 (4.11) | 5.54 (4.27) | <0.001^b^ |
| **Neighborhood education, mean (SD)** | 4.89 (1.91) | 5.25 (2.07) | <0.001^b^ |

*Notes:* HAI, healthy aging index.

^a^ P-value from χ^2^ test.

^b^ P-value from one-way analysis of variance.

**Supplementary Table C3. Association between neighborhood education and HAI levels for participants residing in the same neighborhood for 15 years with imputed data**

|  | HAI | Well-being | Physical and functional health | Sensory health | Mental health |
| --- | --- | --- | --- | --- | --- |
|  | Coef (95% CI) | Coef (95% CI) | Coef (95% CI) | Coef (95% CI) | Coef (95% CI) |
| **Model 1^a^** | | | | | |
| Neighborhood education | 0.75***  (0.61 - 0.89) | 1.73***  (1.53 - 1.94) | 0.54***  (0.34 - 0.74) | 0.80***  (0.49 - 1.10) | 1.15***  (0.86 - 1.44) |
| **Model 2^a^** | | | | | |
| Individual education | 0.35***  (0.30 - 0.40) | 1.12***  (1.05 - 1.20) | 0.33***  (0.26 - 0.39) | 0.26***  (0.15 - 0.36) | 0.36***  (0.25 - 0.46) |
| Neighborhood education | 0.43***  (0.28 - 0.58) | 0.67***  (0.45 - 0.89) | 0.24**  (0.03 - 0.45) | 0.56***  (0.23 - 0.89) | 0.82***  (0.51 - 1.13) |
| **Model 3^a^** | | | | | |
| Neighborhood education | 0.76***  (0.62 - 0.90) | 1.77***  (1.56 - 1.98) | 0.55***  (0.35 - 0.75) | 0.80***  (0.49 - 1.10) | 1.15***  (0.86 - 1.45) |
| Difference between Ind. and Neighbor. Edu.^b^ | | | | | |
| Ind. Edu. = Neighbor. Edu. | 0 | 0 | 0 | 0 | 0 |
| Ind. Edu. < Neighbor. Edu. | -0.98***  (-1.33 - -0.64) | -4.38***  (-4.93 - -3.82) | -1.39***  (-1.94 - -0.85) | -0.22  (-1.04 - 0.59) | -0.54  (-1.42 - 0.34) |
| Ind. Edu. > Neighbor. Edu. | 1.49***  (1.16 - 1.82) | 4.60***  (4.05 - 5.15) | 1.44***  (0.99 - 1.88) | 1.09**  (0.35 - 1.84) | 1.88***  (1.14 - 2.63) |
| **Model 4^a^** | | | | | |
| Individual education | 0.53***  (0.46 - 0.60) | 1.33***  (1.20 - 1.46) | 0.36***  (0.27 - 0.45) | 0.55***  (0.40 - 0.69) | 0.68***  (0.54 - 0.82) |
| Difference between Ind. and Neighbor. Edu. | | | | | |
| Ind. Edu. = Neighbor. Edu. | 0 | 0 | 0 | 0 | 0 |
| Ind. Edu. < Neighbor. Edu. | 0.49***  (0.10 - 0.88) | -0.59**  (-1.19 - 0.01) | -0.27  (-0.85 - 0.31) | 1.12***  (0.26 - 1.97) | 1.47***  (0.55 - 2.39) |
| Ind. Edu. > Neighbor. Edu. | -0.82***  (-1.28 - -0.37) | -1.27***  (-1.94 - -0.61) | -0.05  (-0.64 - 0.53) | -1.32***  (-2.29 - -0.35) | -1.00*  (-2.09 - 0.08) |

*Notes*: HAI, healthy aging index; Coef, coefficient; CI, confidence interval; Ind. Edu., individual education; Neighbor. Edu., neighborhood education.

^a^ Models were adjusted for age, gender, marital status, area of residence, occupation and frequency of drinking, including 37007 observations for each effect size.

^b^ Difference between Ind. and Neighbor. Edu.: individual education equal to, lower, or higher than the neighborhood’s at the time of investigation.

*p < 0.1, **p < 0.05, ***p < 0.01.

**Supplementary Table C4. Association between neighborhood education and HAI trajectories for participants residing in the same neighborhood for 15 years with imputed data**

|  | HAI trajectories^a^ |
| --- | --- |
|  | OR (95% CI) |
| **Model 1^b^** | |
| Neighborhood education | 1.23*** (1.15 - 1.32) |
| **Model 2^b^** | |
| Individual education | 1.13*** (1.10 - 1.16) |
| Neighborhood education | 1.10*** (1.02 - 1.19) |
| **Model 3^b^** | |
| Neighborhood education | 1.23*** (1.17 - 1.32) |
| Difference between Ind. and Neighbor. Edu. during the follow-up period | |
| Ind. Edu. = Neighbor. Edu.^c^ | 1 |
| Ind. Edu. < Neighbor. Edu.^d^ | 0.66*** (0.53 - 0.82) |
| Ind. Edu. > Neighbor. Edu.^e^ | 1.46*** (1.18 - 1.82) |
| Others^f^ | 0.96 (0.68 - 1.36) |
| **Model 4^b^** | |
| Individual education | 1.19*** (1.15 - 1.24) |
| Difference between Ind. and Neighbor. Edu. during the follow-up period | |
| Ind. Edu. = Neighbor. Edu.^c^ | 1 |
| Ind. Edu. < Neighbor. Edu.^d^ | 1.01 (0.81 - 1.27) |
| Ind. Edu. > Neighbor. Edu.^e^ | 0.70** (0.53 - 0.92) |
| Others^f^ | 0.91 (0.64 - 1.30) |

*Notes*: HAI, healthy aging index; OR, odds ratio; CI, confidence interval.

^a^ HAI trajectories were grouped as low starting point and fast decline; median starting point and slow decline; high starting point and slow decline; and high starting point with increase followed by decline. The worst health condition group was regarded as reference group. Includes 9595 observations for each effect size.

^b^ Models were adjusted for age, gender, marital status, area of residence, occupation and frequency of drinking.

^c^ Ind. Edu. = Neighbor. Edu., individual education equal to the neighborhood’s for more than half of the time during follow-up.

^d^ Ind. Edu. < Neighbor. Edu., individual education lower than the neighborhood’s for more than half of the time during follow-up.

^e^ Ind. Edu. > Neighbor. Edu., individual education higher than the neighborhood’s for more than half of the time during follow-up.

^f^ Others, individuals lived in a neighborhood with a changing average education relative to individual education during follow-up.

*p < 0.1, **p < 0.05, ***p < 0.01.

**Supplementary Table C5. Age stratified association between neighborhood education and HAI levels with imputed data**

|  | Under age 65 | Over age 65 |
| --- | --- | --- |
|  | Coef (95% CI) | Coef (95% CI) |
| **Model 1^a^** | | |
| Neighborhood education | 0.76*** (0.58 - 0.93) | 0.73*** (0.57 - 0.88) |
| **Model 2^a^** | | |
| Individual education | 0.42*** (0.34 - 0.49) | 0.31*** (0.21 - 0.40) |
| Neighborhood education | 0.35*** (0.19 - 0.52) | 0.44*** (0.28 - 0.61) |
| **Model 3^a^** | | |
| Neighborhood education | 0.76*** (0.58 - 0.94) | 0.73*** (0.58 - 0.88) |
| Difference between Ind. and Neighbor. Edu.^b^ |  |  |
| Ind. Edu. = Neighbor. Edu. | 0 | 0 |
| Ind. Edu. < Neighbor. Edu. | -0.81*** (-1.22 - -0.40) | -1.02*** (-1.54 - -0.50) |
| Ind. Edu. > Neighbor. Edu. | 2.01*** (1.62 - 2.39) | 1.28*** (0.78 - 1.78) |
| **Model 4^a^** | | |
| Individual education | 0.55*** (0.48 - 0.62) | 0.52*** (0.41 - 0.63) |
| Difference between Ind. and Neighbor. Edu. |  |  |
| Ind. Edu. = Neighbor. Edu. | 0 | 0 |
| Ind. Edu. < Neighbor. Edu. | 0.77*** (0.36 - 1.18) | 0.60** (0.07 - 1.12) |
| Ind. Edu. > Neighbor. Edu. | -0.57*** (-1.04 - -0.10) | -0.79*** (-1.37 - -0.21) |

*Notes*: HAI, healthy aging index; Coef, coefficient; CI, confidence interval; Ind. Edu., individual education; Neighbor. Edu., neighborhood education.

^a^ Models were adjusted for age, gender, marital status, area of residence, occupation and frequency of drinking, including 28774 observations for under age 65 and 15234 for over age 65 for each effect size.

^b^ Difference between Ind. and Neighbor. Edu.: individual education equal to, lower, or higher than the neighborhood’s at the time of investigation.

*p < 0.1, **p < 0.05, ***p < 0.01

**Supplementary Table C6. Age stratified association between neighborhood education and HAI trajectories^a^ with imputed data**

|  | Under age 65 | Over age 65 |
| --- | --- | --- |
|  | OR (95% CI) | OR (95% CI) |
| **Model 1^b^** | | |
| Neighborhood education | 1.25*** (1.16 - 1.35) | 1.19*** (1.08 - 1.31) |
| **Model 2^b^** | | |
| Individual education | 1.17*** (1.12 - 1.21) | 1.13*** (1.06 - 1.19) |
| Neighborhood education | 1.07** (0.99 - 1.17_ | 1.07 (0.96 - 1.20) |
| **Model 3^b^** | | |
| Neighborhood education | 1.26*** (1.15 - 1.37) | 1.20*** (1.09 - 1.31) |
| Difference between Ind. and Neighbor. Edu. during the follow-up period | | |
| Ind. Edu. = Neighbor. Edu.^c^ | 1 | 1 |
| Ind. Edu. < Neighbor. Edu.^d^ | 0.65** (0.49 - 0.87) | 0.71* (0.49 - 1.03) |
| Ind. Edu. > Neighbor. Edu.^e^ | 1.48** (1.03 - 2.11) | 1.82*** (1.25 - 2.65) |
| Others^f^ | 0.74 (0.47 - 1.15) | 0.71 (0.29 - 1.71) |
| **Model 4^b^** | | |
| Individual education | 1.24*** (1.18 - 1.31) | 1.14*** (1.07 - 1.22) |
| Difference between Ind. and Neighbor. Edu. during the follow-up period | | |
| Ind. Edu. = Neighbor. Edu.^c^ | 1 | 1 |
| Ind. Edu. < Neighbor. Edu.^d^ | 1.16 (0.86 - 1.57) | 1.04 (0.71 - 1.54) |
| Ind. Edu. > Neighbor. Edu.^e^ | 0.62** (0.42 - 0.94) | 1.03 (0.64 - 1.67) |
| Others^f^ | 0.70 (0.46 - 1.07) | 0.80 (0.34 - 1.92) |

*Notes*: HAI, healthy aging index; OR, odds ratio; CI, confidence interval.

^a^ HAI trajectories were grouped as low starting point and fast decline; median starting point and slow decline; high starting point and slow decline; and high starting point with increase followed by decline. The worst health condition group was regarded as reference group. Includes 9004 observations for each effect size for under age 65 and 2629 for over age 65.

^b^ Models were adjusted for age, gender, marital status, area of residence, occupation and frequency of drinking.

^c^ Ind. Edu. = Neighbor. Edu., individual education equal to the neighborhood’s for more than half of the time during follow-up.

^d^ Ind. Edu. < Neighbor. Edu., individual education lower than the neighborhood’s for more than half of the time during follow-up.

^e^ Ind. Edu. > Neighbor. Edu., individual education higher than the neighborhood’s for more than half of the time during follow-up.

^f^ Others, individuals lived in a neighborhood with a changing average education relative to individual education during follow-up.

*p < 0.1, **p < 0.05, ***p < 0.01.

**Supplementary Table C7. Association between neighborhood education and HAI levels for complete cases**

|  | HAI | Well-being | Physical and functional health | Sensory health | Mental health |
| --- | --- | --- | --- | --- | --- |
|  | Coef (95% CI) | Coef (95% CI) | Coef (95% CI) | Coef (95% CI) | Coef (95% CI) |
| **Model 1^a^** | | | | | |
| Neighborhood education | 0.80***  (0.60 – 1.00) | 2.25***  (1.89 - 2.61) | 0.41***  (0.24 - 0.58) | 0.84***  (0.48 - 1.19) | 0.84***  (0.54 - 1.13) |
| **Model 2^a^** | | | | | |
| Individual education | 0.38***  (0.30 - 0.45) | 1.22***  (1.14 - 1.31) | 0.25***  (0.20 - 0.31) | 0.32***  (0.20 - 0.44) | 0.35***  (0.24 - 0.47) |
| Neighborhood education | 0.40***  (0.23 - 0.57) | 1.00***  (0.67 - 1.34) | 0.15*  (-0.03 - 0.32) | 0.50***  (0.14 - 0.87) | 0.47***  (0.18 - 0.76) |
| **Model 3^a^** | | | | | |
| Neighborhood education | 0.79***  (0.59 – 1.00) | 2.21***  (1.83 - 2.58) | 0.40***  (0.24 - 0.57) | 0.83***  (0.46 - 1.20) | 0.83***  (0.55 – 1.11) |
| Difference between Ind. and Neighbor. Edu.^b^ | | | | | |
| Ind. Edu. = Neighbor. Edu. | 0 | 0 | 0 | 0 | 0 |
| Ind. Edu. < Neighbor. Edu. | -0.51**  (-0.96 - -0.07) | -4.53***  (-5.27 - -3.80) | -0.88***  (-1.49 - -0.28) | 0.48  (-0.41 - 1.37) | -0.14  (-1.22 - 0.94) |
| Ind. Edu. > Neighbor. Edu. | 1.72***  (1.29 - 2.14) | 4.65***  (3.63 - 5.67) | 1.20***  (0.78 - 1.61) | 1.78***  (0.95 - 2.61) | 2.10***  (1.04 - 3.15) |
| **Model 4^a^** | | | | | |
| Individual education | 0.61***  (0.48 - 0.74) | 1.75***  (1.45 - 2.04) | 0.29***  (0.22 - 0.36) | 0.65***  (0.45 - 0.84) | 0.58***  (0.45 - 0.71) |
| Difference between Ind. and Neighbor. Edu. | | | | | |
| Ind. Edu. = Neighbor. Edu. | 0 | 0 | 0 | 0 | 0 |
| Ind. Edu. < Neighbor. Edu. | 1.22***  (0.67 - 1.77) | 0.43  (-0.29 - 1.14) | -0.05  (-0.60 - 0.51) | 2.32***  (1.25 - 3.40) | 1.50***  (0.39 - 2.61) |
| Ind. Edu. > Neighbor. Edu. | -0.93***  (-1.61 - -0.25) | -2.97***  (-5.01 - -0.92) | -0.07  (-0.58 - 0.44) | -1.03  (-2.07 - 0.02) | -0.41  (-1.55 - 0.73) |

*Notes*: HAI, healthy aging index; Coef, coefficient; CI, confidence interval; Ind. Edu., individual education; Neighbor. Edu., neighborhood education.

^a^ Models were adjusted for age, gender, marital status, area of residence, occupation and frequency of drinking, including 34123 observations for each effect size.

^b^ Difference between Ind. and Neighbor. Edu.: individual education equal to, lower, or higher than the neighborhood’s at the time of investigation.

*p < 0.1, **p < 0.05, ***p < 0.01

**Supplementary Table C8. Association between neighborhood education and HAI trajectories for complete cases**

|  | HAI trajectories^a^ |
| --- | --- |
|  | OR (95% CI) |
| **Model 1^b^** | |
| Neighborhood education | 1.30*** (1.22 - 1.38) |
| **Model 2^b^** | |
| Individual education | 1.13*** (1.10 - 1.16) |
| Neighborhood education | 1.14*** (1.07 - 1.22) |
| **Model 3^b^** | |
| Neighborhood education | 1.30*** (1.22 - 1.40) |
| Difference between Ind. and Neighbor. Edu. during the follow-up period | |
| Ind. Edu. = Neighbor. Edu.^c^ | 1 |
| Ind. Edu. < Neighbor. Edu.^d^ | 0.74*** (0.58 - 0.94) |
| Ind. Edu. > Neighbor. Edu.^e^ | 1.63*** (1.22 - 2.17) |
| Others^f^ | 1.19 (0.87 - 1.62) |
| **Model 4^b^** | |
| Individual education | 1.23*** (1.17 - 1.29) |
| Difference between Ind. and Neighbor. Edu. during the follow-up period | |
| Ind. Edu. = Neighbor. Edu.^c^ | 1 |
| Ind. Edu. < Neighbor. Edu.^d^ | 1.29** (1.03 - 1.61) |
| Ind. Edu. > Neighbor. Edu.^e^ | 0.68** (0.46 – 1.00) |
| Others^f^ | 1.06 (0.78 - 1.43) |

Notes: HAI, healthy aging index; OR, odds ratio; CI, confidence interval.

^a^ HAI trajectories were grouped as low starting point and fast decline; median starting point and slow decline; high starting point and slow decline; and high starting point with increase followed by decline. The worst health condition group was regarded as reference group. Includes 8763 observations for each effect size.

^b^ Models were adjusted for age, gender, marital status, area of residence, occupation and frequency of drinking.

^c^ Ind. Edu. = Neighbor. Edu., individual education equal to the neighborhood’s for more than half of the time during follow-up.

^d^ Ind. Edu. < Neighbor. Edu., individual education lower than the neighborhood’s for more than half of the time during follow-up.

^e^ Ind. Edu. > Neighbor. Edu., individual education higher than the neighborhood’s for more than half of the time during follow-up.

^f^ Others, individuals lived in a neighborhood with a changing average education relative to individual education during follow-up.

*p < 0.1, **p < 0.05, ***p < 0.01.

**Supplementary Figure C1. HAI levels by neighborhood education**

*Notes:* HAI, healthy aging index; Neighbor. Edu., neighborhood education.

**Supplementary Figure C2. HAI trajectories by neighborhood education**

*Notes:* HAI, healthy aging index; Neighbor. Edu., neighborhood education.

**
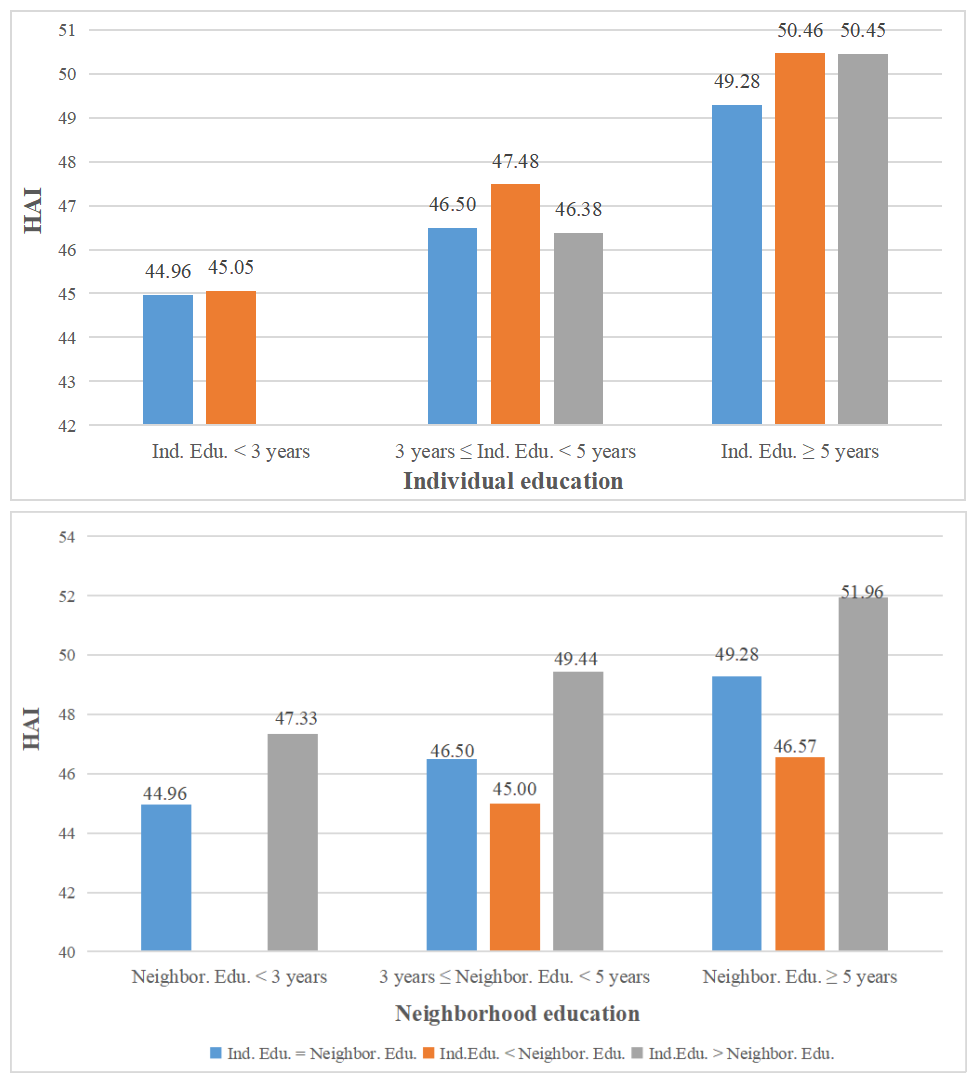
**

**Supplementary Figure C3. HAI levels by neighborhood education and individual education**

*Notes*: HAI, healthy aging index; Neighbor. Edu., neighborhood education; Ind. Edu., individual education.


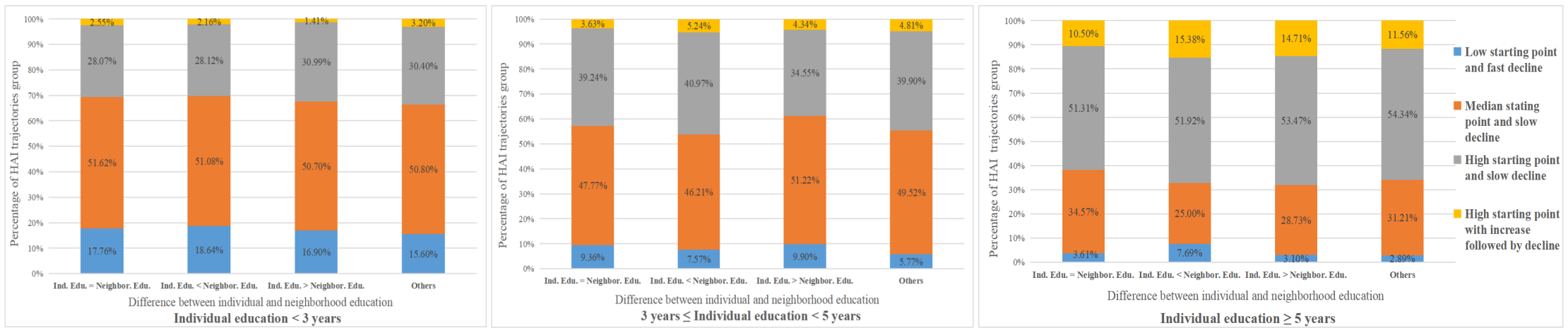


**
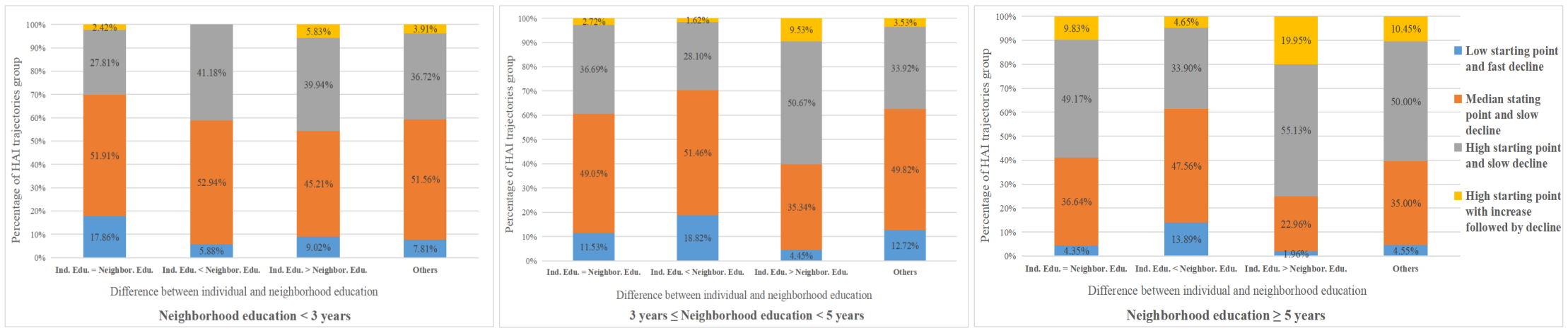
**

**Supplementary Figure C4. HAI trajectories by neighborhood education and individual education**

*Notes*: HAI, healthy aging index; Neighbor. Edu., neighborhood education; Ind. Edu., individual education.

**Section D. Supplementary References**

1. Zhang Z. Multiple imputation with multivariate imputation by chained equation (MICE) package. Ann Transl Med. 2016;4(2):30.
2. World Health Organization. World report on ageing and health. https://apps.who.int/iris/bitstream/handle/10665/186463/9789240694811_eng.pdf?sequence=1. (2015). Accessed November 30, 2021
3. Yang Y, Meng Y. Is China Moving toward Healthy Aging? A Tracking Study Based on 5 Phases of CLHLS Data. Int J Environ Res Public Health. 2020;17(12):4343.
4. Lu W, Pikhart H, Sacker A. Domains and Measurements of Healthy Aging in Epidemiological Studies: A Review. Gerontologist. 2019;59(4):e294-e310.
5. Cheung KS, Lau BH. Successful aging among Chinese near-centenarians and centenarians in Hong Kong: a multidimensional and interdisciplinary approach. Aging Ment Health. 2016;20(12):1314-1326.
6. Whitley E, Benzeval M, Popham F. Associations of Successful Aging With Socioeconomic Position Across the Life-Course: The West of Scotland Twenty-07 Prospective Cohort Study. J Aging Health. 2018;30(1):52-74.
7. Liu LF, Su PF. What factors influence healthy aging? A person-centered approach among older adults in Taiwan. Geriatr Gerontol Int. 2017;17(5):697-707.
8. Paúl C, Ribeiro O, Teixeira L. Active ageing: An empirical approach to the WHO model. Curr Gerontol Geriatr Res. 2012;2012:382972.
9. Lu W, Pikhart H, Sacker A. Comparing socio-economic inequalities in healthy ageing in the United States of America, England, China and Japan: Evidence from four longitudinal studies of ageing. Ageing and Society. 2021;41(7): 1495-1520.
10. Assmann KE, Andreeva VA, Camilleri GM, et al. Dietary scores at midlife and healthy ageing in a French prospective cohort. Br J Nutr. 2016;116(4):666-676.
11. Ran L, X Tan, Xu Y, Zhang K, Zhang Y. The application of subjective and objective method in the evaluation of healthy cities: a case study in Central China. Sustain Cities Soc. 2020; 65: 102581.
12. Caballero FF, Soulis G, Engchuan W, Sánchez-Niubó A, Arndt H, Ayuso-Mateos JL, et al. Advanced analytical methodologies for measuring healthy ageing and its determinants, using factor analysis and machine learning techniques: the ATHLOS project. Sci Rep. 2017;7:43955.
13. Li Y, Aggen S, Shi S, Gao J, Li Y, Tao M, et al. The structure of the symptoms of major depression: exploratory and confirmatory factor analysis in depressed Han Chinese women. Psychol Med. 2014;44(7):1391-1401.

14. Thoma MV, Kleineidam L, Forstmeier S, Maercker A, Weyerer S, Eisele M, et al. Associations and correlates of general versus specific successful ageing components. Eur J Ageing. 2020;18(4):549-563.

15. Yao SS, Cao GY, Han L, Chen ZS, Huang ZT, Gong P, et al. Prevalence and Patterns of Multimorbidity in a Nationally Representative Sample of Older Chinese: Results From the China Health and Retirement Longitudinal Study. J Gerontol A Biol Sci Med Sci. 2020;75(10):1974-1980.

16. Phinyomark A, Osis S, Hettinga BA, Ferber R. Kinematic gait patterns in healthy runners: A hierarchical cluster analysis. J Biomech. 2015;48(14):3897-3904.

17. Askar M, Cañadas RN, Svendsen K. An introduction to network analysis for studies of medication use. Res Social Adm Pharm. 2021;17(12):2054-2061.

18. Liu J, Hao J, Sun Y, Shi Z. Network analysis of population flow among major cities and its influence on COVID-19 transmission in China. Cities. 2021;112:103138.

19. Chen CH. A Hybrid Multi-Criteria Decision-Making Approach Based on ANP-Entropy TOPSIS for Building Materials Supplier Selection. Entropy (Basel). 2021;23(12):1597.

20. Ozkaya G, Erdin C. Evaluation of smart and sustainable cities through a hybrid MCDM approach based on ANP and TOPSIS technique. Heliyon. 2020;6(10):e05052.

21. Nagin DS. Group-based trajectory modeling: an overview. Ann Nutr Metab. 2014;65(2-3):205-210.

22. Hu YH, Halstead MR, Bryan RN, Schreiner PJ, Jacobs DR, Jr SidneyS, et al. Association of Early Adulthood 25-Year Blood Pressure Trajectories With Cerebral Lesions and Brain Structure in Midlife. JAMA Netw Open. 2022;5(3):e221175.
